# A Metaheuristic Optimization Algorithm for Task Clustering in Collaborative Multi-Cluster Systems

**DOI:** 10.3390/s26041364

**Published:** 2026-02-20

**Authors:** Meixuan Li, Yongping Hao, Hui Zhang, Jiulong Xu

**Affiliations:** School of Equipment Engineering, Shenyang Ligong University, Shenyang 110159, China; 2200600015@stu.sylu.edu.cn (M.L.); syluzhanghui@163.com (H.Z.); xujiulong@sylu.edu.cn (J.X.)

**Keywords:** UAV swarm, meta-inspired optimization algorithms, K-means, three-dimensional battlespace, task grouping

## Abstract

To address the task-grouping problem for air–ground integrated Unmanned Aerial Vehicle (UAV) swarm missions in three-dimensional (3D) environments, this study proposes a data-preprocessing and hybrid initialization clustering method based on 3D spatial features. A dual-modal prototype meta-heuristic optimization model, Dual-Prototype Metaheuristic K-Means (DPM-Kmeans), is constructed accordingly. First, to overcome spatial information loss in high-dimensional task allocation, a 3D spatial task data preprocessing technique and a hybrid initialization strategy based on the golden spiral distribution are designed. This ensures the diversity and environmental adaptability of the initial solutions. Second, a dual-modal prototype optimization framework incorporating row prototypes (local refinement) and column prototypes (global combination) was constructed using meta-heuristics and clustering algorithms. The prototype-driven replacement update mechanism simultaneously performs global and local search, balancing the algorithm’s exploration and exploitation capabilities while expanding the solution space. This effectively addresses premature convergence issues in complex search spaces. Simultaneously, a collaborative multi-constraint, dynamically weighted optimization model was constructed, incorporating task requirements and flight distance constraints to ensure that the grouping scheme approximates the global optimum. Simulation results demonstrate that compared to traditional K-means and mainstream meta-heuristic optimization algorithms, DPM-Kmeans achieves an overall improvement of 2–10% in Sum of Squared Errors (SSE), Silhouette Coefficient (SC), and Davies–Bouldin Index (DB) metrics. It exhibits superior convergence speed and solution quality, proving the method’s excellent scalability and robustness in multi-constraint, large-scale 3D scenarios.

## 1. Introduction

With the increasing maturity of drone technology and airborne sensor payloads—such as electro-optical infrared, synthetic aperture radar, and lidar—drones equipped with diverse sensors are being widely deployed in modern intelligent combat, rescue operations, and other fields. In scenarios such as reconnaissance, surveillance, and target tracking, the effectiveness of the swarm is highly dependent on the perception quality and coverage range of the sensor network [1,2]. From the perspective of sensor networks, rational task grouping fundamentally constitutes the collaborative scheduling of finite sensor resources and the optimal spatial partitioning for coverage. Conventional approaches struggle to address issues of sensor data redundancy or perception blind spots in large-scale, dynamic environments, leading to conflicts in sensor resource utilization and energy efficiency wastage. Therefore, in complex environments with multiple constraints, a rational task grouping strategy not only enhances flight stability and autonomous combat and rescue capabilities, but is also crucial for optimizing sensor observation angles, improving the quality of multi-source data fusion, and achieving robust operation of sensor networks [3,4,5].

Task grouping fundamentally involves classifying and categorizing task point information acquired by sensors in space, making clustering algorithms a common approach for solving such problems. By mining multi-source sensor data streams, clustering methods achieve efficient partitioning of task areas. In recent years, as research in data mining has deepened, numerous optimized clustering algorithms have emerged, including metaheuristic, swarm intelligence, and game-based algorithms. Among these, heuristic methods have become indispensable tools for achieving balanced optimization of sensor perception efficiency and energy consumption in dynamically complex environments, owing to their adaptability and effectiveness [6].

In 3D environments, the optimization problem of UAV mission grouping typically exhibits complex characteristics such as high dimensionality, nonlinearity, and multimodality. This renders traditional mathematical programming methods inefficient or even infeasible [7]. Against this backdrop, meta-heuristic algorithms can obtain high-quality approximate optimal solutions within reasonable computational time, offering a viable and efficient alternative [8,9]. For instance, relevant research has significantly optimized system energy efficiency and response latency in UAV edge computing offloading and multi-constraint task processing, respectively, through an improved hybrid natural heuristic algorithm and a clustering-based scheduling strategy [10,11]. Such algorithms demonstrate tremendous potential in handling complex, large-scale, and nonlinear problems, highlighting their advantages over traditional deterministic methods.

### 1.1. Related Work

Large-scale complex tasks executed by multi-UAV coordination have transitioned from conceptual research to practical application. However, challenges such as expanding mission volume and spatial dimensions, along with diversifying mission requirements, have made it difficult for traditional rule-based approaches or single algorithms to strike a balance between efficiency, feasibility, and robustness [12]. Although researchers have proposed various optimization- and heuristic-based approaches to enhance local performance, most studies focus on incremental algorithmic improvements or validation within constrained scenarios. There remains a lack of systematic methodologies and comprehensive scalability evaluations tailored for large-scale 3D environments. Given this, there is an urgent need for a hierarchical, hybrid processing strategy that preserves spatial information and allocation quality to support real-time or near-real-time scheduling for complex tasks. Based on this starting point, the following sections will elaborate on problem modeling, key technical bottlenecks, and the proposed solution and validation approach.

#### 1.1.1. Task Allocation in UAV Swarms

Researchers widely employ various intelligent optimization algorithms to address challenges in task allocation in UAV swarms. References [13,14,15,16,17] respectively employ networked evolutionary games, K-means clustering optimization, multi-objective evolutionary algorithms, and other meta-heuristic algorithms to address the trade-off between computational efficiency and load balancing in dynamic, complex environments. These methods enhance global search capabilities by increasing population diversity, demonstrating significant advantages in optimization efficiency and solution quality while reducing reliance on traditional heuristic rules. As documented in [13], the improved Grey Wolf Optimizer (GWO) is applied to heterogeneous multi-UAV task allocation. In [14], a multi-objective evolutionary algorithm is employed to address the task allocation problem. These studies provide research directions for task allocation in complex scenarios. In [17], the task allocation problem for reconnaissance missions targeting heterogeneous objectives is addressed by proposing an HTRA allocation scheme that integrates an enhanced particle swarm optimization algorithm with gradient projection methods. Through the collaborative optimization of task sequences and reconnaissance timing, this approach significantly enhances the reconnaissance effectiveness and mission completion rate of unmanned aerial vehicle (UAV) swarms.

#### 1.1.2. Task Allocation in High-Dimensional and Complex Environments

Unlike traditional 2D task assignment, real-world environments are typically 3D, featuring significant variations in task intensity, time/energy constraints, and platform capability heterogeneity. For high-dimensional task allocation problems, Reference [18] addresses communication redundancy in decentralized clusters by proposing a Clustering-CBBA algorithm. Through a hierarchical conflict resolution mechanism, it significantly reduces communication overhead while ensuring global benefits for large-scale task execution. Reference [19] employs 3D region coverage and pheromone diffusion techniques (decomposing three-dimensional space into multiple two-dimensional planes). This approach reduces decision complexity and computational dimensions while achieving efficient, robust task allocation for drone swarms, alleviating computational challenges under multi-objective conditions. Although these methods alleviate computational pressure under multi-objective conditions to some extent, they still face challenges such as the curse of dimensionality, computational overhead, and partial loss of critical spatial information when performing global optimization on massive task points within high-dimensional data. Reference [20] achieves joint optimization of path and task allocation for UAV swarms in complex 3D environments by integrating an improved RRT-APF path planning algorithm with adaptive potential fields and global search, alongside a discrete particle swarm task assignment algorithm. This approach significantly reduces computational overhead.

#### 1.1.3. Improvements and Challenges in Optimization Algorithms

Regarding the task allocation and optimization problem for large-scale UAV swarms, existing research can be broadly categorized into two types. The first type focuses on the task allocation model itself, employing sophisticated algorithms to solve complex combinatorial optimization problems. For instance, Reference [21] proposes an improved self-organizing mapping (ISOM) algorithm incorporating an attention mechanism. This approach effectively enhances the execution efficiency and flexibility of dynamic reallocation problems in large-scale task environments for drone swarms while maintaining load balancing. Reference [22] proposes a Probabilistic Chain Enhanced Parallel Genetic Algorithm (PC-EPGA), combined with Bayesian network-optimized parameters, to effectively enhance the solution quality and computational efficiency for task allocation and sequence planning in large-scale UAV fleets. Reference [23] addresses multi-UAV coordinated strike operations in naval warfare by proposing a two-stage greedy auction algorithm using information entropy weighting, significantly improving the real-time performance and stability of task allocation for large-scale UAV swarms. Reference [24] addresses the task reallocation problem in multi-UAV cooperative reconnaissance by proposing a two-stage distributed task allocation algorithm (TS-DTA). By comprehensively considering performance differences and dynamic constraints such as time windows, it achieves efficient task recovery while significantly reducing the communication overhead of the swarm. The second category transforms the task allocation problem into a clustering process, such as the approach in [25] that combines K-Means++ with a genetic algorithm (GA). First, K-Means++ resolves the initialization sensitivity issue by grouping regions, followed by GA for global path and task optimization, significantly reducing computational complexity. Reference [26] synergistically optimizes deployment schemes and power allocation for multi-UAV-assisted NOMA systems through K-means clustering and an improved particle swarm optimization (PSO) algorithm, substantially enhancing system energy efficiency. To overcome the sensitivity to initial centers and susceptibility to local optima in traditional clustering algorithms like K-means, Reference [27] introduced K-means clustering via an improved IALNS (Iterative Adaptive Large Neighborhood Search) to generate initial solutions. Combined with a simulated annealing acceptance criterion, this approach enhances solution diversity and escapes local optima.

These methods have improved solution efficiency and the ability to escape local optima to some extent, but they still face challenges such as insufficient coverage of the initial population, reduced search efficiency in later stages, and high sensitivity to hyperparameters. To address the initialization challenge, hybrid initialization strategies have proven effective in balancing solution coverage with alignment to task distributions.

### 1.2. Motivations and Contributions

The aforementioned research has advanced the development of UAV task allocation technology in various aspects, but the following challenges remain:

- As the number of UAVs and the scale of missions increase, particularly when handling high-dimensional task allocation, a “dimension disaster” may occur. This leads to the loss of topological information regarding sensor-perceived targets, causing sensor vision scheduling to deviate from actual requirements.
- The phenomenon of “premature convergence” in heuristic algorithms leads to stagnation and suboptimal solutions, becoming more severe in high-dimensional and deceptive search space. These algorithms struggle to balance exploration and exploitation, rendering them incapable of locating global optima under complex perceptual constraints such as sensor resolution and overlap rate requirements.
- Existing task allocation architectures are primarily designed for specific or single task types, lacking the collaborative capabilities and versatility required for multi-task and multi-scenario environments.

In summary, to construct a universal and scalable task grouping architecture for 3D environments that addresses the challenges of poor optimization performance in existing algorithms under large-scale and multi-scenario conditions, this paper proposes a two-stage allocation method. This approach combines hybrid clustering for pre-grouping with meta-heuristic re-optimization, aiming to enhance both the efficiency of task grouping in complex three-dimensional environments and the search coverage capabilities of sensor nodes within three-dimensional space. The specific contributions are as follows:

- To address the high spatial complexity of 3D tasks, a clustering grouping method integrating 3D data preprocessing with a hybrid initialization strategy based on the golden spiral distribution is proposed. This approach effectively preserves critical information such as elevation and terrain, thereby enhancing the perception coverage and spatial adaptability of sensor networks within complex three-dimensional environments.
- We propose the DPM-Kmeans meta-heuristic optimization model based on K-means, constructing dual-modal prototypes—row-wise prototypes and column-wise prototypes. The two prototypes respectively undertake local refinement tasks (such as minimizing spatial deviation between sensors and targets to enhance observation quality) and global combinatorial search tasks (improving sensor coverage uniformity and reducing observation redundancy), operating in parallel through a prototype-driven update mechanism. This approach accelerates convergence while expanding the solution space, enhancing the algorithm’s ability to escape local optima.
- Based on mission requirements, flight distance, and UAV quantity constraints, a multi-constraint comprehensive weighted optimization model was constructed. Through the synergistic interaction of linear and nonlinear weighting functions, the model ensures that grouping schemes satisfy all constraints while approximating the global optimal solution.

## 2. Problem Description

**Figure 1:**
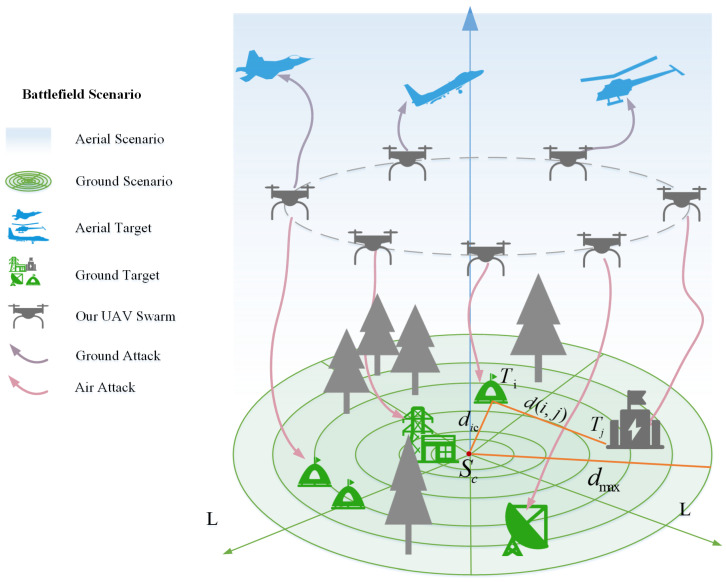
Task Scenario Diagram.

In the 3D task space L, there are *n* discrete task points $T=T_{1},\ldots ,T_{n}$, each task point $T_{i}$ perceived by environmental sensors (such as airborne or ground-mounted radar, electro-optical/infrared sensors, LiDAR, etc.) and possessing three-dimensional coordinates $q_{i}=\left(x_{i},y_{i},z_{i}\right)$ alongside a task requirement intensity $r_{i}$. The n task points must be partitioned into k task groups $S=S_{1},\ldots ,S_{k}$ to enable their collaborative execution by a drone swarm equipped with diverse sensors. The objective is to optimize grouping to enhance mission execution efficiency while satisfying constraints such as flight distance, resources, and payload balancing. This problem is essentially a multi-constraint resource scheduling and topology optimization issue for mobile sensor networks, driven by three-dimensional spatial features. Figure 1 illustrates a typical scenario where a drone swarm utilizes its onboard sensors to perform collaborative operations within a three-dimensional task space. Leveraging the data acquisition capabilities of these sensors, the swarm can flexibly address diverse real-world operational requirements, including environmental surveys, post-disaster monitoring, and infrastructure inspections, while Table 1 provides definitions for sets, parameters, and variables.

To ensure each task is assigned to only one group and avoid resource wastage, design task assignment constraints and decision variables $x_{ij}=\left\{0,1\right\}$. When $x_{ij}$ = 1, it indicates task $T_{i}$ has been assigned to task group $S_{j}$; otherwise, $x_{ij}$ = 0. Establish the task assignment constraints as follows:

(1) $$\sum_{i\in n}x_{ij}=1,\;\forall j\in k\;$$

Given the varying demand across target tasks, the decision variable $x_{ij}$ is used to screen and calculate demand for each grouped task. This ensures that the demand for each group strictly equals the total demand for its assigned tasks, without exceeding the predetermined maximum value. The constraints are as follows:

(2) $$\;r_{\max}\geq \sum_{i=1}^{k}z_{i},\;z_{i}=\sum_{i=1}^{n}r_{i}\cdot x_{ij},\;\forall j\in k$$

Here, $r_{i}$ represents the demand for the *i*-th task, and $z_{j}$ represents the total task demand for the *j*-th grouping cluster.

In a 3D combat environment, to prevent excessive dispersion of mission points that can delay UAV responses, increase energy consumption, or exceed operational ranges, a maximum distance constraint is introduced: each mission point’s Euclidean distance from its group center must not exceed the $d_{\max}$ threshold.

(3) $$\;d_{ic}\leq d_{\max},\;\forall i\in n$$

Here, $d_{ic}$ represents the Euclidean distance between task point *i* and its group center *c*.

To ensure the feasibility of scheduling plans under limited resource conditions and prevent execution conflicts caused by over-allocation, the total number of UAVs allocated must not exceed the system’s available resources. The UAV quantity constraints are as follows:

(4) $$\sum_{i=1}^{k}u_{i}\leq m$$

Here, $u_{i}$ represents the number of UAVs assigned to the *i*-th group, and *m* denotes the total number of UAVs available in the system.

## 3. Hybrid Initialization Clustering-Based Grouping

To simultaneously enhance the coverage, data adaptability, and diversity of the initial population in clustering, a hybrid initialization strategy is proposed. The initial population is proportionally composed of geometrically uniform samples based on the golden circular distribution (ensuring coverage of the solution space) and data-driven samples generated by task-point clustering mapping (aligning with the true distribution). This enables the algorithm to rapidly enter meaningful search regions, avoid getting stuck in noise, and strengthen its ability to escape local optima.

### 3.1. Data Preprocessing

In high-dimensional spatial tasks, traditional methods often suffer from severely degraded clustering performance due to data sparsity; conversely, overly simplified modeling strategies result in the loss of spatial structural information, making it difficult to meet practical requirements. To address these challenges, domain-specific clustering optimization methods are employed to reduce spatial complexity while fully preserving spatial structural features.

To ensure the stability of cluster centers and the overall quality of spatial partitioning, the task demand intensity $r_{j}$ is first incorporated into the distance metric to construct a weighted distance, thereby defining the nth-order weighted neighborhood $N_{n}\left(i\right)$ (see Equation (5)). Based on this weighted neighborhood, a local density $lrd_{\omega ,n}\left(i\right)$ is proposed (see Equation (6)) to quantify the spatial clustering of task points, thus preventing high-demand tasks from being misclassified as outliers due to low neighborhood density. Subsequently, the task-aware outlier factor $lof_{\omega ,n}\left(i\right)$ (see Equation (7)) is introduced. By comparing the density estimation deviation between task point $T_{i}$ and points within its neighborhood, and setting a threshold ω, points with deviations exceeding ω are classified as dense points, while those falling below ω are identified as outliers. Parameter $d_{n}\left(i\right)$ determines the scope of the weighted neighborhood.

(5) $$N_{n}\left(i\right)=\left\{T_{j}|d\left(i,j\right)\left(1+\frac{r_{j}}{r_{\max}}\right)\leq d_{n}\left(i\right)\right\}$$

(6) $${\mathrm{lrd}}_{\omega ,n}\left(i\right)=\left(1+\frac{r_{i}}{r_{\max}}\right)\sum_{l\in N_{n}\left(i\right)}\frac{|N_{n}\left(i\right)|}{\max\left(d_{n}\left(i\right),d_{\omega }\left(i,j\right)\right)}$$

(7) $${\mathrm{lof}}_{\omega ,n}\left(i\right)=\frac{\sum_{j\in N_{n}\left(i\right)}{\mathrm{lrd}}_{\omega ,n}\left(j\right)}{{\mathrm{lrd}}_{\omega ,n}\left(i\right)\cdot N_{n}\left(i\right)}$$

**Figure 2:**
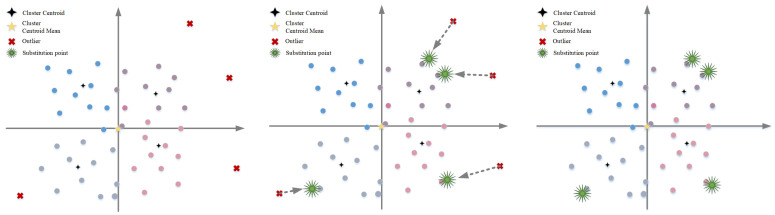
Schematic diagram of outlier replacement method.

To avoid discarding outliers that may contain critical information, a virtual point substitution strategy is proposed (see Figure 2): For each outlier, a virtual point of the same type is first generated at its nearest dense point, and then the original task’s requirement information is fully mapped to this virtual point. The grey dashed arrows in the figure illustrate the substitution process. This method preserves all task requirements while minimizing spatial perturbations, thereby ensuring the stability of the clustering structure and maintaining task semantics consistency.

### 3.2. Hybrid Initialization Strategy

**Figure 3:**
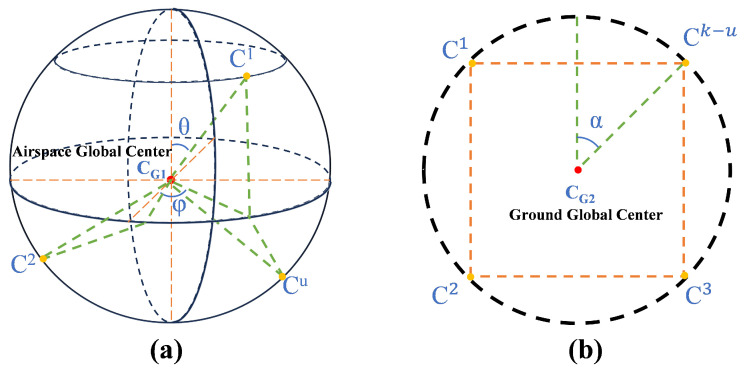
Defining cluster centers. (**a**) Aerial scene $G_{1}$. (**b**) Ground scene $G_{2}$.

Traditional clustering methods employ non-spatial clustering (2D clustering logic). Unlike conventional 2D modeling approaches (e.g., Reference [19]), this paper addresses 3D spatial characteristics by dividing the global 3D space into aerial scene $G_{1}$ and ground scene $G_{2}$. It then utilizes the golden spiral distribution strategy to define cluster centers for each scene. The golden spiral distribution on a 3D sphere naturally aligns with the spherical structure of 3D space, and the distribution of cluster centers conforms to the geometric properties of 3D space rather than being forcibly extended into three dimensions (as in planar grid distributions). Figure 3a,b show the geometric configurations of the cluster centers in $G_{1}$ and $G_{2}$, respectively.

We employ golden spiral initialization to generate an approximate low-discrepancy point distribution on the sphere. This distribution provides more uniform initial spatial coverage when domain prior knowledge is lacking, reducing the probability of initialization producing clusters or voids. Consequently, it facilitates the population’s early global exploration and diminishes the risk of becoming trapped in local optima. Compared to random distributions in 3D space, it exhibits greater isotropy and uniform coverage, thereby preventing initial solutions from becoming trapped in local minima.

Under the aerial scene $G_{1}$, to achieve optimal spatial coverage uniformity within a finite sensing volume, we employ the golden ratio constant ϕ to construct a quasi-random sequence (Fibonacci Lattice). This selection method offers the advantage of generating point distributions exhibiting extremely low discrepancy and isotropic characteristics. Specifically, within the scene, *u* points must be uniformly distributed across the sphere, numbered i=1,2,…,u, and the sphere’s center is defined as $C_{G_{1}}=\left(\frac{X}{2},\frac{Y}{2},\frac{Z}{2}\right)$. Based on the golden spiral distribution strategy, the golden ratio constant $\varphi =\frac{1+\sqrt{5}}{2}$ is introduced to sequentially calculate the polar angle $\theta_{i}$ and azimuth angle $\phi_{i}$ for the *i*-th point (see Equation (8)).

(8) $$\theta_{i}=\arccos\left(1-2\cdot \frac{i-1}{u-1}\right),\;\phi_{i}=\frac{2\pi (i-1)}{\varphi }$$

These two angles jointly determine the position of point *i* on the sphere, achieving uniform distribution of sampling points and avoiding local overcrowding or voids. Based on this, the 3D coordinates $C_{G_{1}}^{i}=\left(C_{X}^{i},C_{Y}^{i},C_{Z}^{i}\right)$ of the *i*-th point can be expressed as:

(9) $$C_{X}^{i}=\frac{X}{2}+R\cdot \sin\left(\theta_{u}\right)\cdot \cos\left(\phi_{u}\right),\;C_{Y}^{i}=\frac{Y}{2}+R\cdot \sin\left(\theta_{u}\right)\cdot \sin\left(\phi_{u}\right),\;C_{Z}^{i}=\frac{Z}{2}+R\cdot \cos\left(\theta_{u}\right)$$

In ground scene $G_{2}$, to achieve uniform distribution of k−u cluster centers along the circumference of a 2D plane, define the center point $C_{G_{2}}=\left(X/2,Y/2\right)$ and assign point indices s=1,2,…,k−u. To avoid the initial position coinciding with the coordinate axes, an offset angle α is introduced. In this paper, α is set to π/4. The coordinates of the *s*-th cluster center are given by Equation (10). Subsequently, all task points are grouped according to the nearest cluster center, yielding the grouping scheme. This approach ensures that ground-based unmanned aerial vehicles can be uniformly deployed along a circular perimeter at the outset, forming a layered, overlapping coverage pattern with aerial sensing nodes.

(10) $$C_{X}^{s}=\frac{X}{2}+\frac{X_{\max}}{4}\cdot \sin\left(\frac{2\pi \left(s-(u+1)\right)}{k}+\alpha \right),\;C_{Y}^{s}=\frac{Y}{2}+\frac{Y_{\max}}{4}\cdot \cos\left(\frac{2\pi \left(s-(u+1)\right)}{k}+\alpha \right)$$

To obtain diverse initial solutions, a golden spiral distribution strategy is employed to generate a set of reference cluster centers on the target sphere. Leveraging the rotational properties of 3D space, random rotation matrices from the special orthogonal group SO(3) are applied to transform these reference cluster centers. Rotation axes and angles are randomly generated n times, yielding n sets of cluster centers. Finally, the K-means algorithm was applied to group task points into k clusters. This was then blended with the results from the golden spiral method to form a complete population as the initial population, thereby avoiding the “noise solutions” caused by fully random initialization.

## 4. Dual-Prototype Metaheuristic K-Means

To address the issue of meta-heuristic optimization algorithms easily getting stuck in local optima and exhibiting low search efficiency in complex solution spaces, a dual prototype construction approach is proposed. This approach assigns distinct responsibilities to the two prototypes: one handles local refinement, while the other performs global combinatorial search. The walking prototype, which serves as a representative of the behavior of “real individuals,” is used during the exploitation phase to replace the worst individual or to perform local refinements on candidate solutions. This accelerates convergence and steadily enhances solution quality. Prototype generation, by extracting typical value distributions for each task point, is employed during the exploration phase. It generates numerous new combinations through concatenation to escape local optima and maintain population diversity.

### 4.1. Dual Prototype Construction

To address premature convergence and local optima in meta-heuristic algorithms, Reference [16] proposed a K-means Optimizer (KO) optimization algorithm based on a stochastic approach and a population-based method. This method employs the K-means clustering algorithm and fundamental mathematical equations to devise two distinct motion strategies, enabling search agents to roam freely within suboptimal regions to identify potential search spaces, thereby achieving the capability to escape local optima. However, this method only clusters and reorganizes the columns of the candidate solution matrix to identify a more optimal exploration space. The DPM-Kmeans method builds upon this by also clustering the rows of the candidate solution matrix to achieve a balance between exploitation and exploration.

DPM-Kmeans is a population-based approach that solves problems or performs optimization by generating a matrix composed of multiple candidate solutions. It utilizes the N×M matrix $P=p_{1},p_{2},\cdots ,p_{N}$ formed from the initial solutions generated in Section 3, as shown in Equation (11), where *N* is the population size. Each row $P_{i}$ (with *M* variables) represents a grouping scheme.

(11) $$P=\left\{\begin{array}{c} P_{1}\\ P_{2}\\ P_{3}\\ \vdots \\ P_{N} \end{array}\right\}={\left\{\begin{array}{cccc} P_{1,1} & P_{1,2} & \ldots & P_{1,M}\\ P_{2,1} & P_{2,2} & \ldots & P_{2,M}\\ P_{3,1} & P_{3,2} & \ldots & P_{3,M}\\ \vdots & \vdots & \vdots & \vdots \\ P_{N,1} & P_{N,2} & \ldots & P_{N,M} \end{array}\right\}}^{N\times M}$$

After obtaining the matrix *P*, the fitness of each individual $p_{i}$ is calculated using the objective function MinF, yielding the fitness matrix F(P) (Equation (12)). To achieve the goal of finding optimal solutions and attaining rapid convergence, the quality of the search space is described by F(P). Only search spaces with the potential to yield high-quality solutions are considered, while those of inferior quality are disregarded.

(12) $$F\left(P\right)={\left\{\begin{array}{c} {\mathrm{Fitness}}_{1}=\mathrm{MinF}\left(P_{1}\right)\\ {\mathrm{Fitness}}_{2}=\mathrm{MinF}\left(P_{2}\right)\\ {\mathrm{Fitness}}_{3}=\mathrm{MinF}\left(P_{3}\right)\\ \vdots \\ {\mathrm{Fitness}}_{n}=\mathrm{MinF}\left(P_{N}\right) \end{array}\right\}}^{N\times 1}$$

Subsequently, the fitness matrix is combined with matrix *P* as a weighting condition, and the individuals are rearranged in descending order of fitness to form the weighted matrix $P_{w}$, as shown in Equation (13).

(13) $$P_{\omega }={\left\{\begin{array}{ccccc} P_{1,1} & P_{1,2} & \ldots & P_{1,M} & {\mathrm{Fitness}}_{1}\\ P_{2,1} & P_{2,2} & \ldots & P_{2,M} & {\mathrm{Fitness}}_{2}\\ P_{3,1} & P_{3,2} & \ldots & P_{3,M} & {\mathrm{Fitness}}_{3}\\ \vdots & \vdots & \vdots & \vdots & \vdots \\ P_{N,1} & P_{N,2} & \ldots & P_{N,M} & {\mathrm{Fitness}}_{N} \end{array}\right\}}^{N\times (M+1)}$$

#### 4.1.1. Row-Wise Clustering

Due to the distinct discrete characteristics in the representation of individual solutions, high-quality solutions often exhibit similar grouping patterns. If these patterns can be extracted, they can not only filter out noise generated by random mutations but also guide the population toward the center of high-potential search regions. Therefore, this paper employs row-wise clustering to group populations at the row vector level, aiming to accelerate exploitation by extracting “population consensus.”

During clustering, apply the K-modes method to assign each column (task point dimension) a centroid component based on the most frequently occurring group label in each cluster. This maintains semantic consistency and feasibility in the extracted prototype solutions. If tied mode values occur, select the label of the current global optimal individual to balance diversity and rational decision-making. The implementation process is as follows:

Initially, the number of clusters $K_{r}$ for row-vector clustering was specified. Initialization was performed using a K-modes heuristic. For each individual solution vector $P_{i}=P_{i,1},P_{i,2},\cdots ,P_{i,M}$ in the population, the distance to each cluster centroid was computed. Given the discrete nature of the task grouping scheme (i.e., discrete labels), the Hamming distance was adopted as the similarity measure, and based on the minimum distance principle, $P_{i}$ is assigned to its corresponding cluster. Within each cluster $C_{r}$, label frequencies were counted per column (task-point dimension). The most frequent label in each column was selected as the centroid component for that column, thus producing the prototype vector R (see Equation (14)), in which *v* represents the grouping scheme of $P_{i}$.

(14) $$R_{k}\left[j\right]=\arg\max_{v\in \{1,2,3\}}\sum_{i\in C_{k}}1\left(P_{i,j}=v\right),\;J=1,\ldots ,M$$

This step ensures that the proto vector represents a feasible grouping scheme. The assignment and update steps were iteratively repeated until the cluster partitioning no longer changed or a maximum number of iterations was reached. Finally, *K* representative prototype vectors $R=R_{1},R_{2},\cdots ,R_{K}$ were obtained.

(15) $$P\left(t\right)\overset{K\text{-}\mathrm{modes}}{\to }R\left(t\right)=\left\{\begin{array}{c} R_{1}\left(t\right)\\ R_{2}\left(t\right)\\ R_{3}\left(t\right)\\ \vdots \\ R_{K}\left(t\right) \end{array}\right\}={\left\{\begin{array}{cccc} R_{1,1}^{t} & R_{1,2}^{t} & \ldots & R_{1,M}^{t}\\ R_{2,1}^{t} & R_{2,2}^{t} & \ldots & R_{2,M}^{t}\\ R_{3,1}^{t} & R_{3,2}^{t} & \ldots & R_{3,M}^{t}\\ \vdots & \vdots & \vdots & \vdots \\ R_{K,1}^{t} & R_{K,2}^{t} & \ldots & R_{K,M}^{t} \end{array}\right\}}^{N\times M}$$

#### 4.1.2. Column-Wise Clustering

Simple row clustering tends to result in loss of population diversity. To address this issue, this paper performs column-wise clustering on the task point dimension. The distribution of a single task point across different individuals reveals its latent adaptation tendencies. By reorganizing these tendencies, entirely new solution structures that never appeared in the parent generation can be generated. This enhances the algorithm’s global exploration capabilities, enabling it to escape local optima traps. The process is as follows:

For each task point j=1,…,D, its grouping labels across all individuals were collected to form a vector v(j). Using the K-means algorithm, $K_{c}$ cluster centers G were obtained, yielding the column centroids $c_{1,j},\cdots ,c_{K_{c},j}$. he most frequent label within each cluster was selected as its representative, and the grouping label values were discretely mapped. Finally, the $K_{c}$ representative values of each column were concatenated to form a 3×M matrix *C*. This paper sets $K_{c}$ to 3. Since each column’s values fall within one of three categories 1, 2, 3, setting K=2 would discard one possible assignment, while setting K=4 would result in empty clusters or duplicate centroids. Therefore, setting column-wise *K* equal to the actual number of groups (=3) is the most reasonable approach.

(16) $$P\left(t\right)\overset{K\text{-}\mathrm{means}}{\to }C\left(t\right)=\left\{\begin{array}{c} C_{1}\left(t\right)\\ C_{2}\left(t\right)\\ C_{3}\left(t\right) \end{array}\right\}={\left\{\begin{array}{ccccc} C_{1,1}^{t} & C_{1,2}^{t} & C_{1,3}^{t} & \ldots & C_{1,M}^{t}\\ C_{2,1}^{t} & C_{2,2}^{t} & C_{2,3}^{t} & \ldots & C_{2,M}^{t}\\ C_{3,1}^{t} & C_{3,2}^{t} & C_{3,3}^{t} & \ldots & C_{3,M}^{t} \end{array}\right\}}^{3\times M}$$

**Figure 4:**
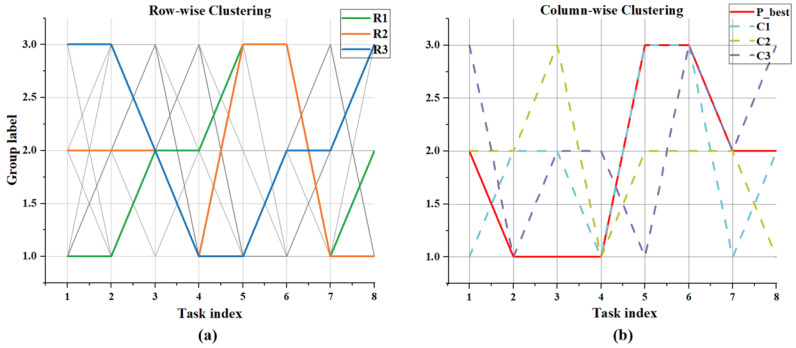
Schematic of dual-prototype clustering. (**a**) Row-wise Clustering. (**b**) Column-wise Clustering.

Figure 4 illustrates the clustering results of Row-wise Clustering versus Column-wise Clustering under conditions of 8 tasks divided into three groups of 10 candidate solutions. Figure 4a: The gray lines represent the 10 candidate solutions generated by the current population, with each candidate solution corresponding to a complete grouping scheme. Colored lines represent the row-wise prototypes ($R_{1},R_{2},R_{3}$) obtained after clustering, which embody the consensus solutions within each cluster. These are used to replace the worst-performing individuals to accelerate convergence. Figure 4b shows the red line representing the global optimal solution $P_{best}$, while the colored lines denote the centroid vectors $C_{1}$, $C_{2}$, and $C_{3}$ extracted from columns. As illustrated, the colored lines gradually converge toward $P_{best}$ during iteration without overlapping with $P_{best}$, thereby avoiding local optima. This demonstrates how exploration introduces new candidate solutions through the typical allocation pattern of task points in the combinatorial task.

### 4.2. Dual-Prototype Update Mechanism

After obtaining the row prototype R(t) and column prototype C(t), a dual-mode parallel update method is employed to achieve local refinement and global exploration of the solution space, as follows.

First, based on the weighted matrix $P_{w}$ obtained from the three sections, select the N solutions p with the worst fitness as candidate replacement solutions. Compare them with the prototype $R_{k}$; if the prototype outperforms the candidate solution, replace the candidate solution to ensure the continuous retention and diffusion of representative patterns within the population. Additionally, to prevent getting stuck in local optima, neighborhood searches are performed on prototypes to generate neighborhood solutions and assess their fitness. If the fitness value surpasses that of weaker individuals in the current population, replacement is also executed to fully explore the high-quality solution space.

Second, after updating the row prototype, concatenate it with the column prototype $C_{k}$ to form a new individual, periodically inserting it into the population. Additionally, to maintain population diversity, partial column crossover is performed between the current best solution $p_{best}$ and column prototype $C_{k}$, generating new solutions that combine global representativeness with the characteristics of the current optimum. After evaluating the fitness of all candidate individuals generated from column prototypes, those that outperform the current worst solution are replaced in the population.

**Figure 5:**
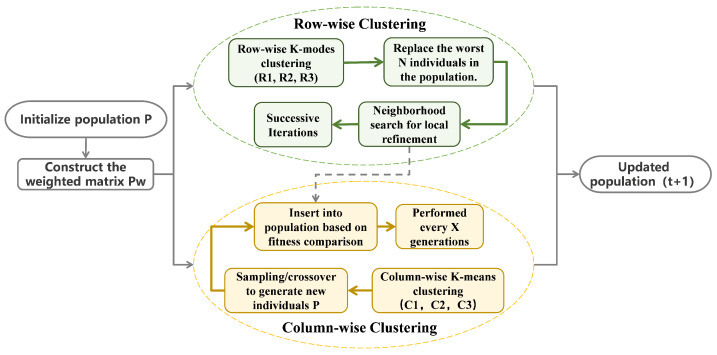
Flowchart of the Dual-Prototype Update Mechanism.

This process effectively combines global exploration with local exploitation, preventing the population from getting stuck in local optima while ensuring the continuous diffusion of high-quality patterns. During iteration, row prototype updates occur sequentially to ensure the population converges toward high-quality solutions, while column prototype updates are performed every τ generations (τ=5 in this paper) to prevent premature convergence. The overall implementation process is illustrated in Figure 5.

Building on the dual-mode update, this paper introduces the LPSR strategy to dynamically regulate population size. As shown in Equation (17), the population size decreases linearly with the iteration count, where t denotes the current iteration count, $T_{max}$ denotes the maximum iteration count, and *N* denotes the initial population size. The minimum population size during the convergence phase is set to n+1 to ensure sufficient solution space.

(17) $$N\left(t\right)=N-\left(N-\left(n+1\right)\right)\cdot \frac{t-1}{T_{\max}-1}$$

In summary, the DPM-Kmeans model achieves a dynamic equilibrium between exploitation and exploration by combining row-prototype-driven local refinement with column-prototype-assisted diversity insertion. This approach enhances the algorithm’s convergence efficiency and global optimization capabilities.

## 5. Experimental Verification

To ensure the fairness of the comparative experiments, KO, PSO-Kmeans, and the proposed DPM-Kmeans were evaluated under identical experimental conditions. Specifically, all algorithms were run on the same hardware platform, employing a uniform initialization strategy (the hybrid initialization described in the Methods section of the paper). Each algorithm was configured with an identical population size = 30 and a maximum of 50 iterations, thereby ensuring equivalence in the total number of fitness function evaluations. The hyperparameters for each algorithm were obtained through identical tuning procedures. Performance metrics for comparison included final fitness, SSE, SC, DB, convergence speed, and average runtime. These settings ensured comparability between methods and enhanced the reliability of conclusions.

### 5.1. Construction of Scenario Data and Evaluation Metric Functions

#### 5.1.1. Scene Construction and Data Generation

To bridge the gap between idealized mathematical models and real-world engineering constraints, this paper establishes a joint simulation verification framework based on Unity3D (version 2022.3.2) and MATLAB (version 2022b). This framework is designed not only to address the limitations of traditional mathematical models in expressing three-dimensional spatial constraints but also to establish the physical environment and data interfaces for subsequent Hardware-in-the-Loop (HIL) experiments in this research. All parameter settings within the simulation platform—including coordinate systems, sampling frequencies, and entity properties—strictly adhere to the real-time performance and engineering feasibility requirements of the HIL system.

**Figure 6:**
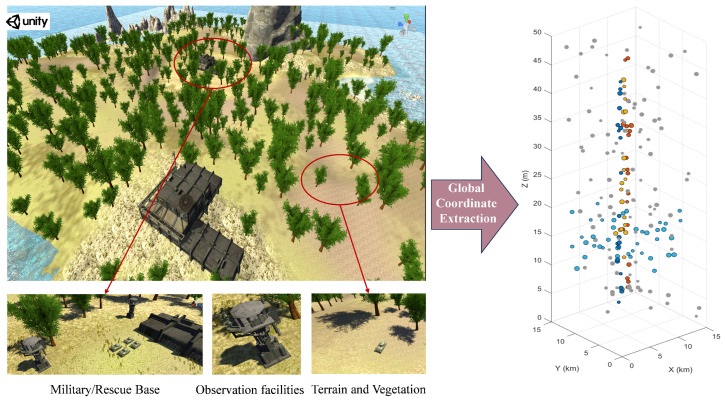
Generate simulation scene diagrams for task points.

Firstly, utilizing Unity3D, a remote wilderness monitoring and search area scenario was constructed. The scene design transcended the limitations of traditional ground-based, single-dimensional approaches, establishing a dual-layer task model encompassing both aerial and ground-level scenarios. The scene spans 15 km by 15 km. Terrain elevation ranging from 0 to 50 m was mapped based on a real-world Digital Elevation Model (DEM), incorporating undulating topography, vegetation occlusion, and key tactical entities such as military bases and watchtowers (as shown in Figure 6). Different coloured dots represent mission types of varying priority. Unlike simple mathematical scatter points, in ground mission scenarios, task points are generated by combining the physics engine with sensor models: Unity’s raycasting mechanism simulates sensor lines of sight (e.g., simulating line laser pulses from airborne cameras or LiDAR), ensuring candidate points actually lie on the surface of three-dimensional terrain or the edges of target entities. At the aerial mission layer, static aerial relay mission points and high-altitude environmental monitoring sampling points are deployed within the 5 m to 50 m altitude airspace. These aerial targets exist as three-dimensional spatial voxels, simulating the spatial coordinates that drones must cover when functioning as mobile communication relay stations or high-altitude sensing nodes.

Secondly, to align with the non-uniform distribution characteristics of mission points in realistic search-and-rescue and tactical scenarios, thereby validating the DPM-Kmeans algorithm’s capability to handle complex spatial topologies, this paper employs an environment-driven probabilistic shift generation model rather than simple uniform random sampling. Specifically, in the Unity scene, predefined key facilities such as bases and watchtowers serve as hotspot centers. Based on these hotspots, high-density clusters are generated through sampling using a Gaussian mixture model (GMM). Simultaneously, mission sequences are sampled using a linear distribution to simulate targets distributed along roads or pathways. To ensure consistency between generated task points and the 3D terrain and sensor observations, Unity’s raycasting is employed to project downward from the generated candidate points. Hit points resulting from valid collisions with terrain or object surfaces are retained, and their world coordinates are written into the dataset. When exporting the database to MATLAB, Unity 3D employs a left-handed coordinate system (*Y*-axis upward), whereas MATLAB and common UAV dynamics models typically use a right-handed coordinate system (*Z*-axis upward). This paper utilizes a transformation matrix to convert the task point coordinates exported from Unity 3D to match the right-handed coordinate system convention.

Additionally, to investigate the algorithm’s performance across different scales, three task sets were constructed with scales of *n* = 200, 300, and 500. Each task sample includes three-dimensional Euclidean coordinates (*x*, *y*, *z*) and physical property features, ensuring realistic relevance in spatial distribution, density, and constraint conditions. The final export of CSV data was imported into the MATLAB platform for algorithm validation. By comparing key metrics such as the fitness function with baseline algorithms, this study verifies the performance advantages of the proposed algorithm in complex engineering environments.

#### 5.1.2. Evaluation Metric Function Construction

To rationally evaluate the merits of candidate grouping schemes, the fitness function was constructed with the dual objectives of maximizing task completion rate and overall reward. This approach holistically considers three key targets: task execution distance, load balancing, and utilization of UAV/sensor resources. Weighting coefficients α,β, and γ were applied to quantify distance costs, demand deviation, and resource utilization, respectively. As both drone energy consumption and sensor energy consumption are critical determinants of mission success during task execution, the energy consumption weighting coefficients α,β, and γ are set to 0.4, 0.3, and 0.3, respectively.

In energy consumption modeling, the “distance cost” within the fitness function not only represents the propulsion energy expenditure incurred by flight distance but also includes sensor energy consumption associated with observation tasks (such as the operational energy expenditure of airborne LiDAR/cameras and the computational overhead of data processing). To minimize overall energy consumption and execution time, the cost function is defined as the sum of Euclidean distances from each task point to its assigned cluster center, where $q_{i}$ denotes the coordinates of the *i*-th task point, $c_{j}$ represents the geometric center of cluster *j*, and $S_{j}$ is the set of tasks within cluster *j*.

(18) $$D=\sum_{j=1}^{k}\sum_{i\in S_{j}}∥q_{i}-c_{j}∥$$

To prevent uneven UAV task distribution caused by excessive load in certain groups, the deviation between each group’s task count and the average task count is used as the load-balancing metric. Here, $\left|S_{j}\right|$ denotes the number of task points within group *j*, *n* represents the total number of tasks, and *k* indicates the number of groups.

(19) $$R=\sum_{j=1}^{k}\left|\left|S_{j}\right|-\frac{n}{k}\right|$$

To enhance resource utilization, the number of UAVs actually deployed is defined as:

(20) $$U=\sum_{j=1}^{k}u_{j},\;u_{j}=\left\{\begin{array}{cc} 1, & \left|S_{j}\right|>0\\ 0, & \left|S_{j}\right|=0 \end{array}\right.$$

Finally, to ensure comparability among the objectives, the three metrics above were normalized individually. Here, $D_{max}$ denotes the maximum task distance in the initial population, while *n* and *k* represent the total number of tasks and the number of groups, respectively.

(21) $$D_{f}=\frac{D}{D_{\max}},\;R_{f}=\frac{R}{n},\;U_{f}=\frac{U}{k}$$

Ultimately, the composite weighted fitness function is expressed as:

(22) $$MinF=\alpha D_{f}+\beta R_{f}+\gamma U_{f}$$

Through the above design, the fitness function can reduce overall consumption while ensuring task execution efficiency, achieve load balancing to prevent group overload, and decrease the number of UAVs used to improve resource utilization.

In this study, the performance of different algorithms is evaluated using the SSE, SC, and DB indices. The Sum of Squared Errors (SSE) is employed to measure the intra-cluster homogeneity. It is defined as the sum of squared distances between each point and the centroid of its corresponding cluster. For K clusters, with cluster $C_{k}$ having centroid $\mu_{k}$, see Equation (23) for the SSE expression. A smaller SSE value indicates superior clustering performance. From a sensor perspective, a smaller SSE corresponds to a shorter average sensor-to-target distance, typically signifying higher detection probability and improved positional accuracy.

(23) $$SSE=\sum_{k=1}^{K}\sum_{x\in C_{k}}{∥x-\mu_{k}∥}^{2}.$$

The Silhouette Coefficient (SC) is employed to evaluate both intra-cluster compactness and inter-cluster separation. The SC value is within the range of [−1, 1], where values close to 1 indicate well-clustered samples, values around 0 suggest samples lying near cluster boundaries, and values close to −1 imply possible misclassification. SC reflects the degree of observation overlap and redundancy between clusters in perception tasks: a higher SC indicates clearer cluster structure, enabling more targeted allocation of sensor resources and reducing redundant coverage. a(i) denotes the average distance between sample *i* and all other points within the same cluster, while b(i) represents the minimum average distance between sample *i* and all other clusters.

(24) $$S\left(i\right)=\frac{b(i)-a(i)}{\max\{a(i),b(i)\}}$$

The Davies–Bouldin Index (DB Index) measures the ratio of within-cluster scatter to between-cluster distance. A smaller value for the DB Index indicates better clustering performance and separation. To calculate the index, it first requires defining the scatter $s_{i}$ of each cluster, as well as the relative similarity $d_{ij}$ between clusters (as presented in Equation (25)). A smaller DB value indicates lower relative dispersion within clusters and better separation between clusters, corresponding to a more distinguishable distribution of sensing tasks and reduced observational interference or conflicts.

(25) $$s_{i}=\frac{1}{\left|C_{i}\right|}\sum_{x\in C_{i}}∥x-\mu_{i}∥,\;d_{ij}=\frac{s_{i}+s_{j}}{M_{ij}}$$

where $M_{ij}=∥\mu_{i}-\mu_{j}∥$ denotes the distance between the centroids of clusters *i* and *j*. Finally, the DB index is defined as the average of the maximum $d_{ij}$ values for each cluster with respect to its most similar cluster:

(26) $$DB=\frac{1}{K}\sum_{i=1}^{K}\max_{j\neq 1}d_{ij}$$

### 5.2. Task Grouping in Multiple Scenarios

To validate the effectiveness, optimality, and scalability of the DPM-Kmeans model, this paper conducted multi-dimensional comparisons and ablation experiments. The benchmark algorithms selected include the population-based K-Means optimizer KO (K-means Optimizer) [16], the meta-heuristic optimizer PSO-Kmeans [28], and the traditional non-swarm benchmark clustering method K-Means [29]. Among these, the KO algorithm, as a swarm optimization search mechanism based on K-Means principles, primarily evolves through row prototype mechanisms; PSO-Kmeans, meanwhile, serves as a typical meta-heuristic optimization benchmark applied to clustering objective functions. Furthermore, to evaluate the core contribution of the dual-prototype synergistic mechanism, an NCM (No Column-wise Mechanism) ablation variant was constructed. NCM retains only the column prototype mechanism as a representative of the ‘Pure Exploration’ strategy, designed to contrast with the full model and validate the role of row prototypes in local refinement. The comparison metrics encompass the SSE, SC, and DB indices alongside the composite fitness function. The maximum iteration count for all algorithms was uniformly set at 50 iterations to ensure experiments were conducted under identical computational resource constraints.

#### 5.2.1. Performance Evaluation of Golden Spiral-Based Initialization Strategy

**Figure 7:**
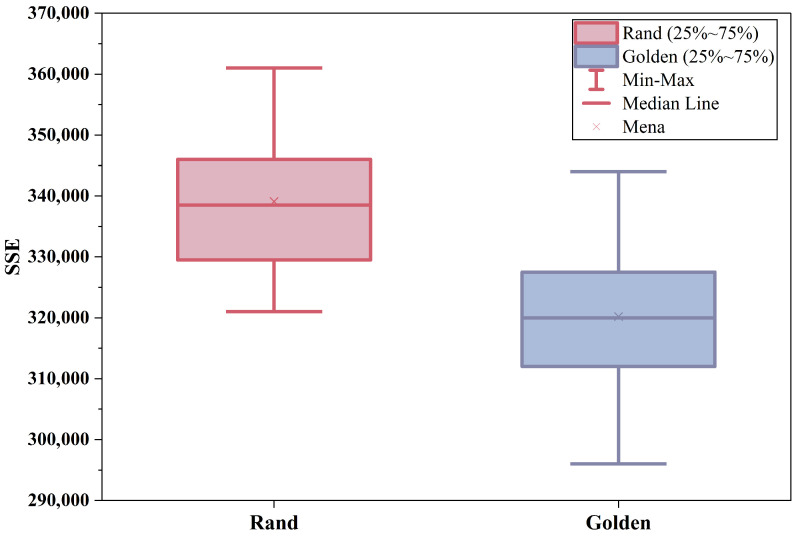
Performance Comparison of Initialization Methods.

To assess the sensitivity and robustness of the initialization method based on the golden spiral, we conducted 20 independent comparative trials against the standard random initialization scheme. The results are shown in Figure 7. Experimental results demonstrate that following initialization with the golden spiral mapping, the SSE is reduced by approximately 6.49% on average. The golden ratio ensures isotropic distribution of the initial cluster centers within 3D space, thereby preventing spatial clustering of sensor nodes. The standard deviation of the golden spiral initialization (13,725.99) was approximately 21% lower than that of the random initialization (17,324.84), indicating that random initialization exhibits high randomness, whereas golden spiral initialization yields more stable clustering results.

The aforementioned results collectively demonstrate that the golden spiral-based initialization strategy can provide more comprehensive and stable initial solutions for subsequent DPM-Kmeans optimization without increasing computational complexity, thereby enhancing the overall quality of task grouping.

#### 5.2.2. Comparison of Experimental Results and Evaluation of Algorithm Convergence Efficiency

To validate algorithm performance, 7, 10, and 17 clusters were respectively set for 200, 300, and 500 task points. Task points were grouped using K-means, KO, NCM, PSO-Kmeans, and DPM-Kmeans models, with a unified evaluation metric employed to quantitatively compare the performance of each algorithm.

**Figure 8:**
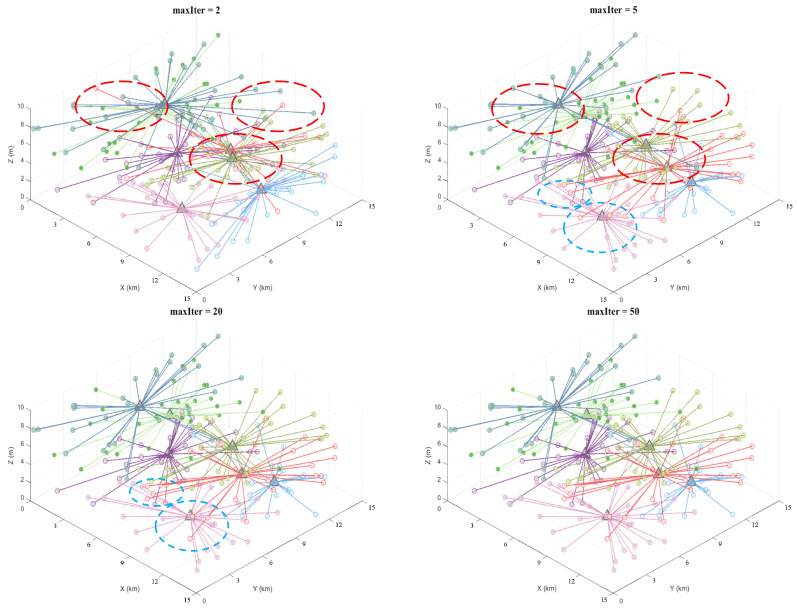
DPM-Kmeans results for 200 task points at iterations 2, 5, 20, and 50.

**Figure 9:**
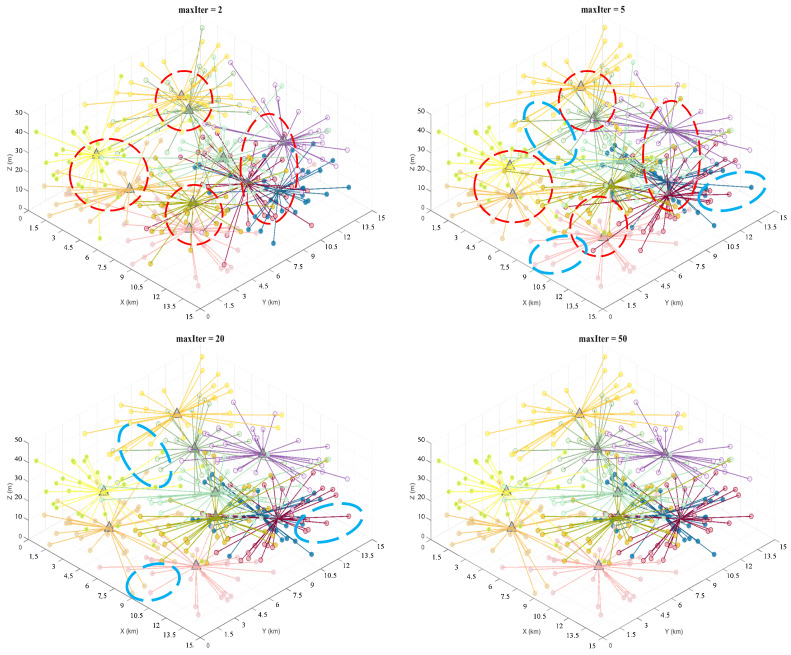
DPM-Kmeans results for 300 task points at iterations 2, 5, 20, and 50.

**Figure 10:**
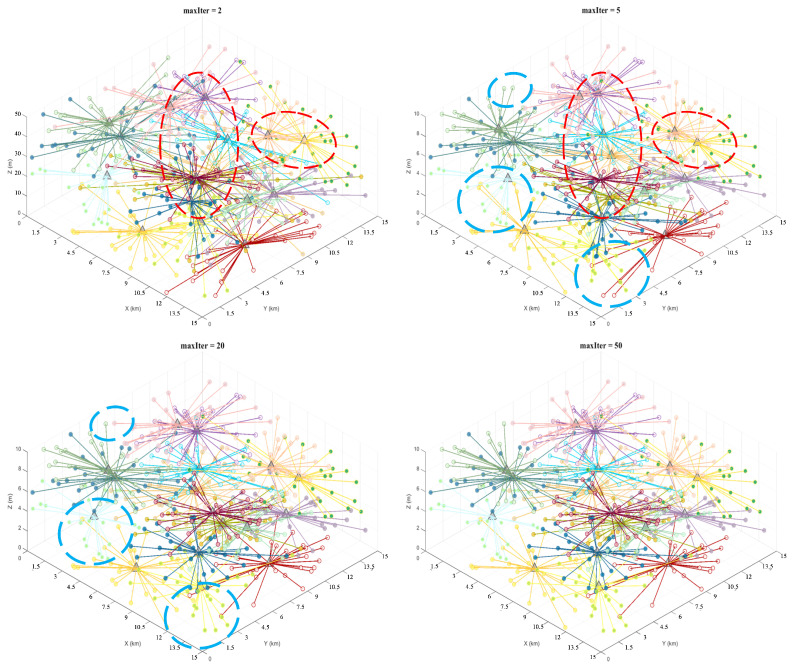
DPM-Kmeans results for 500 task points at iterations 2, 5, 20, and 50.

Figure 8, Figure 9 and Figure 10 illustrate the iterative process for different numbers of task groupings: small circles represent task points, with different colors corresponding to each cluster group, while triangles denote cluster centers. As shown in the figure, the 5th iteration shows gradual improvements over the 2nd iteration in both cluster centers and task-point grouping (indicated by red dashed circles). By the 20th iteration, the cluster centers were largely fixed compared to the 5th iteration, with only minor adjustments to task point groupings (indicated by the blue dashed circles in the figure). The results between the 20th and 50th iterations showed minimal variation, indicating that the clustering results converged and remained stable after approximately 20 iterations. The results indicate that, despite the increased complexity of the proposed model, it converged after 20 iterations with a convergence speed comparable to that of the traditional K-means algorithm, thereby validating the model’s efficiency and applicability in large-scale task grouping scenarios.

**Figure 11:**
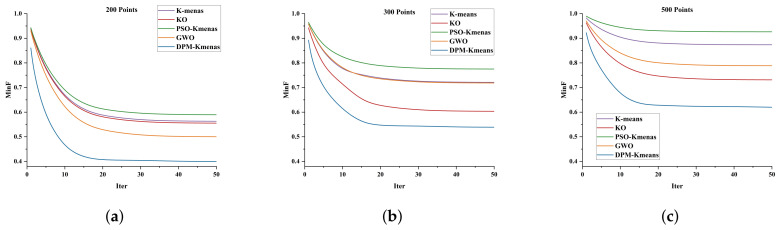
Comparison of fitness values. (**a**) Comparison of Fitness Values at 200 Task Points. (**b**) Comparison of Fitness Values at 300 Task Points. (**c**) Comparison of Fitness Values at 500 Task Points.

The fitness function value serves as a crucial metric for evaluating the quality, convergence efficiency, and stability of solutions obtained by algorithms within the search space. Figure 11 illustrates how the fitness values of DPM-Kmeans and other algorithms evolve over iterations at different task scales. It can be observed that DPM-Kmeans consistently achieves superior fitness function values across all scales compared to other algorithms, while converging faster and exhibiting less fluctuation. This demonstrates that the method outperforms the comparison algorithms in both search efficiency and stability.

#### 5.2.3. Algorithm Performance Comparison

**Figure 12:**
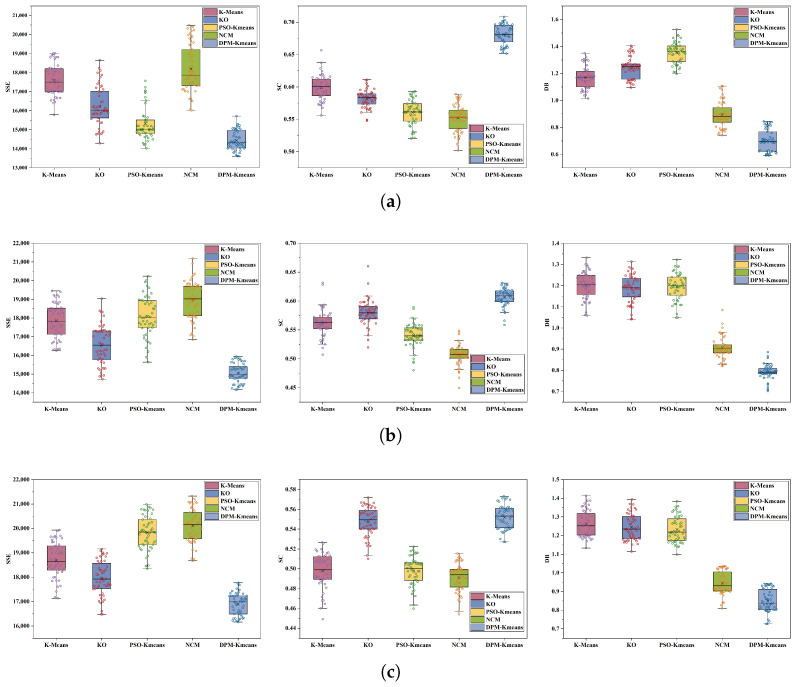
Comparison of objective-function values under different task settings. (**a**) Comparison of objective function values for 200 task points. (**b**) Comparison of objective function values for 300 task points. (**c**) Comparison of objective function values for 500 task points.

Figure 12 and Table 2 present comparisons between DPM-Kmeans and benchmark algorithms (K-means, KO, NCM, PSO-Kmeans) across 50 independent experiments at varying scales. The mean values in the numerical results reflect the algorithms’ average search capability, while the standard deviation indicates robustness under random initialization. DPM-Kmeans maintains a superior mean while keeping the standard deviation within an acceptable range, demonstrating its overall reliability in task allocation.

#### 5.2.4. Validation of the Effectiveness of Scene Segmentation Methods

The data was divided into 2D segments with each plane spaced 2 m apart. Tasks were grouped into sets of 100, 200, 300, 400, 500, and 600 to validate the effectiveness of the domain-based clustering optimization model. Table 3 presents the mean results, comparing the average values of various metrics (SSE, SC, DB) across the two scenarios.

**Figure 13:**
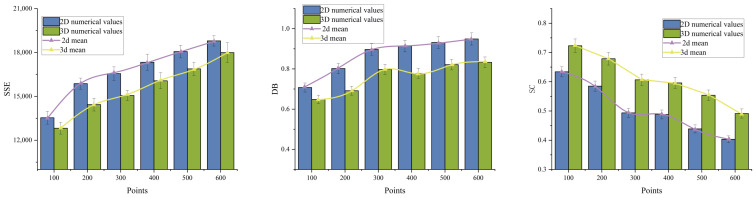
Comparison of various indicators (SSE, SC, DB) in 2D and 3D scenes.

Figure 13 presents the comparison results of SSE, SC, and DB metrics across two scenarios. Compared to the 2D slicing approach, direct modeling of the entire 3D environment fully captures vertical spatial relationships, enabling more effective height-level segmentation. This avoids the need for additional merging and layer-level adjustments in 2D models, thereby achieving lower SSE values. Simultaneously, during the cluster optimization process, precise spatial distance metrics increased between-cluster variance while significantly reducing within-cluster variance, resulting in the DB metric performing markedly better than in 2D scenarios.

### 5.3. Computational Complexity Analysis

The computational efficiency of the DPM-Kmeans algorithm depends on the number of tasks N, the number of clusters K, the population size P, the number of iterations T, and the dimension D. Although the hybrid initialization and dual prototype construction phase incurs some computational overhead, its magnitude is significantly lower than that of the update and evaluation phases. The update and evaluation phases require evaluating the fitness of P individuals using the Euclidean distance over T iterations, with each evaluation having complexity O(N·K·D). Therefore, the overall runtime of the algorithm is dominated by the iterative optimization process, with a total time complexity of O(T·P·N·K·D). In contrast, benchmark algorithms (PSO-Kmeans, NCM, KO) are all population-based algorithms. Their structure involves iterating T times × a population of P individuals × calculating the fitness of each individual. Since the fitness calculation remains O(N·K·D), their overall complexity is O(T·P·N·K·D).

In summary, compared to the traditional K-means algorithm’s computational complexity of O(T·N·K·D), DPM-Kmeans introduces the population factor P to achieve global optimization, resulting in higher computational costs. However, compared to other meta-heuristic algorithms, DPM-Kmeans maintains the same order of complexity (O(T·P·N·K·D)). This demonstrates that the additional computational overhead introduced by the dual-prototype clustering mechanism is negligible, ensuring that DPM-Kmeans remains computationally competitive.

## 6. Conclusions

This paper proposes the DPM-Kmeans optimization model to address challenges such as suboptimal performance and poor adaptability in traditional clustering models when tackling UAV swarm task allocation problems. The DPM-Kmeans model employs a hybrid initialization and dual-mode replacement framework, utilizing hybrid initialization solutions as seeds. Through prototype-driven replacement, splicing, and sampling, it achieves targeted expansion of the solution space and accelerated convergence. By preserving the synergy between retention and generation mechanisms, the approach balances steady knowledge improvement with diversity maintenance, thereby enhancing both the ability to escape local optima and the robustness of the final partition. The two prototypes are designed to work in parallel and in tandem, enabling the algorithm to continuously generate new search directions while maintaining stable convergence. Finally, under multiple constraints, including mission requirements, flight distance, and UAV quantity, a comprehensive weighted optimization model was constructed. Numerical simulation experiments demonstrated the method’s outstanding performance in complex and large-scale mission scenarios, while ablation studies further validated the model’s stability and robustness in 3D environments. In summary, the DPM-Kmeans optimization model provides an efficient, scalable, and stable solution for task allocation in UAV swarms within 3D environments, demonstrating significant theoretical significance and practical value.

## Figures and Tables

**Table 1:** Main Notation.

Symbol	Description
$T=\{T_{1},\ldots ,T_{n}\}$	Task set, *n* is the total number of tasks
$S=\{S_{1},\ldots ,S_{k}\}$	Task grouping set, *k* is the number of groups
$G=\{G_{1},G_{2}\}$	Scene set, $G_{1}$ denotes aerial scenes and $G_{2}$ denotes ground scenes;
$P=\{P_{1},\ldots ,P_{n}\}$	Candidate Solution Population Set
$q_{i}=\left(x_{i},y_{i},z_{i}\right)$	3D coordinate of task point $T_{i}$
*m*	Total number of UAVs
$r_{i}$	UAV requirement for task point $T_{i}$
$z_{j}$	UAV requirements for Subgroup $S_{j}$, $z_{j}=\sum_{i\in S_{j}}r_{i}$
$r_{\max}$	Maximum UAV demand
$u_{j}$	Actual number of UAVs used in group $S_{j}$
$c_{j}$	Geometric center (centroid) of group $S_{j}$
$d_{i,c}$	Euclidean distance from task point $T_{i}$ to task grouping center $c_{c(i)}$
d(i,j)	Distance from task point $T_{i}$ and $T_{j}$
$d_{\max}$	Maximum distance between the mission point and $c_{j}$
*D*	Maximum flight distance for UAVs
*L*	Spatial scope

**Table 2:** Results of mean and standard deviation.

200	K-Means	KO	PSO-Kmeans	NCM	DP-Kmeans
SSE	17,590 ± 794	16,238 ± 1016	15,316 ± 1031	18,203 ± 1363	14,436 ± 517
SC	0.5976 ± 0.0178	0.5815 ± 0.0164	0.5588 ± 0.0261	0.5543 ± 0.0269	0.6804 ± 0.0283
DB	1.1746 ± 0.0812	1.2558 ± 0.0778	1.3669 ± 0.0941	0.8911 ± 0.0982	0.6969 ± 0.0885
300	K-Means	KO	PSO-Kmeans	NCM	DP-Kmeans
SSE	17,732 ± 913	16,644 ± 1204	17,995 ± 1117	18,920 ± 1248	15,108 ± 812
SC	0.5657 ± 0.0254	0.5763 ± 0.0273	0.5407 ± 0.0252	0.5065 ± 0.0203	0.6078 ± 0.0213
DB	1.2028 ± 0.0714	1.1897 ± 0.0703	1.1965 ± 0.0709	0.9140 ± 0.0506	0.7915 ± 0.0416
500	K-Means	KO	PSO-Kmeans	NCM	DP-Kmeans
SSE	18,754 ± 819	18,017 ± 793	20,005 ± 868	20,386 ± 830	16,906 ± 558
SC	0.4968 ± 0.0204	0.5473 ± 0.0177	0.4960 ± 0.0174	0.4883 ± 0.0161	0.5502 ± 0.0154
DB	1.2726 ± 0.0813	1.2545 ± 0.0788	1.2427 ± 0.0786	0.9672 ± 0.0634	0.8519 ± 0.0710

**Table 3:** Results of mean in 2D and 3D scenarios.

Points	Index	2D	3D	Points	Index	2D	3D
	SSE	13,538	12,822		SSE	15,869	14,436
100	SC	0.6340	0.7230	200	SC	0.5849	0.6787
	DB	0.7075	0.6484		DB	0.8019	0.6914
	SSE	16,559	15,060		SSE	17,331	16,065
300	SC	0.4936	0.6065	400	SC	0.4887	0.5960
	DB	0.8968	0.7976		DB	0.9147	0.778
	SSE	18,062	16,879		SSE	18,793	17,993
500	SC	0.4387	0.5539	600	SC	0.4033	0.4912
	DB	0.9316	0.8213		DB	0.9491	0.8331

## Data Availability

The raw data supporting the conclusions of this article will be made available by the authors on request.

## Abbreviations

The following abbreviations are used in this manuscript:

DPM-Kmeans	Dual-Prototype Metaheuristic K-Means
KO	K-Means Optimization
PSO-Kmeans	Particle Swarm Optimization K-Means
NCM	No column-wise clustering mechanism
SSE	Sum of Squared Errors
DB	Davies–Bouldin Index
SC	Silhouette 20 Coefficient
UAV	Unmanned Aerial Vehicle
